# Procedural sedation for direct current cardioversion: a feasibility study between two management strategies in the emergency department

**DOI:** 10.1186/s12872-020-01664-1

**Published:** 2020-08-25

**Authors:** Giulia Stronati, Alessandro Capucci, Antonio Dello Russo, Erica Adrario, Andrea Carsetti, Michela Casella, Abele Donati, Federico Guerra

**Affiliations:** 1 Cardiology and Arrhythmology Clinic, Marche Polytechnic University, University Hospital “Ospedali Riuniti Umberto I - Lancisi - Salesi”, Via Conca 71, Ancona, Italy; 2 Anaesthesia and Intensive Care Unit, Marche Polytechnic University, University Hospital “Ospedali Riuniti”, Ancona, Italy

**Keywords:** Atrial fibrillation, Cardioversion, Emergency procedures, Midazolam, Propofol, Sedation

## Abstract

**Background:**

A cardiologist-only approach to procedural sedation with midazolam in the setting of elective cardioversion (DCC) for AF has already been proven as safe as sedation with propofol and anaesthesiologist assistance. No data exist regarding the safety of such a strategy during emergency procedures. The aim of this study is to compare the feasibility of sedation with midazolam, administered by a cardiologist, to an anaesthesiologist-assisted protocol with propofol in emergency DCC.

**Methods:**

Single centre, prospective, open blinded, randomized study including all consecutive patients admitted to the Emergency Department requiring urgent or emergency DCC. Patients were randomized in a 1:1 fashion to either propofol or midazolam treatment arm. Patients in the midazolam group were managed by the cardiologist only, while patients treated with propofol group underwent DCC with anaesthesiologist assistance.

**Results:**

Sixty-nine patients were enrolled and split into two groups. Eighteen patients (26.1%) experienced peri-procedural adverse events (bradycardia, severe hypotension and severe hypoxia), which were similar between the two groups and all successfully managed by the cardiologist. No deaths, stroke or need for invasive ventilation were registered. Patients treated with propofol experienced a greater decrease in systolic and diastolic blood pressure when compared with those treated with midazolam.

As the procedure was shorter when midazolam was used, the median cost of urgent/emergency DCC with midazolam was estimated to be 129.0 € (1st-3rd quartiles 114.6–151.6) and 195.6 € (1st-3rd quartiles 147.3–726.7) with propofol (*p* < .001).

**Conclusions:**

Procedural sedation with midazolam given by the cardiologist alone was feasible, well-tolerated and cost-effective in emergency DCC.

## Background

Direct current cardioversion (DCC) represents the most widely used and effective method to restore sinus rhythm in patients with persistent atrial fibrillation (AF) [1–3]. DCC is, however, a painful procedure that can cause pain comparable to a surgical incision [4]. It therefore requires both analgesia and deep sedation which, according to the American Society of Anaesthesiologists, consists in a drug induced reduction of the level of consciousness, during which patients can respond purposefully to painful stimuli [5]. The use of sedation during DCC can also play a role in reducing the pain-related catecholamine surge therefore preventing the recollection of such an unpleasant experience by the patient [6, 7].

To this day, no specific guidelines or recommendations with regards to the most appropriate drug that should be used for procedural sedation is available and different sedation strategies have been described in literature but no consensus on the most efficient strategy has been reached. One of the most commonly used agent for sedation in DCC, is propofol [8] an intravenous sedative hypnotic drug which can be administered only by personnel trained in advanced airways management [9]. In order to overcome such a limitation, non-anaesthesiologists, such as cardiologists, emergency physicians or nurses [10] have sought potential alternatives such as benzodiazepines. In particular, the use of a cardiologist-only approach to sedation with midazolam for elective DCC has been described in literature [11, 12] and demonstrated to be as tolerated and effective as propofol in elective procedures [13]. However, concerns have been raised on the use of a cardiologist-only, midazolam-based strategy in urgent or emergency DCC as haemodynamic instability and lack of time could precipitate the risk of stroke and serious adverse events [9, 14].

The aim of this prospective, open-blinded, randomized study is to compare the feasibility of sedation with midazolam, administered exclusively by a cardiologist, to an anaesthesiologist-assisted protocol with propofol in urgent or emergency DCC.

## Methods

### Population and inclusion criteria

The “Intravenous beNzodiazepine Safety and Tolerability in Emergency Atrial fibrillation Direct-current cardioversion” (INSTEAD) is a single centre, prospective, open blinded, randomized study included consecutive patients admitted to the Emergency Department or the University Hospital “Ospedali Riuniti Umberto I - Lancisi - Salesi” requiring urgent or emergency DCC. The present study follows a pilot trial [14] that has already been performed in order to assess clinical feasibility.

Emergency DCC was defined as DCC performed in haemodynamically compromised patients with new-onset AF, according to European guidelines [1]. Urgent DCC was defined as DCC performed within 2 h from patient’s admission due to persistent invalidating symptoms such as chest pain, pre-syncope, or severe palpitations. No anti-arrhythmic drugs were given prior to DCC for pharmacological cardioversion.

The inclusion criteria consisted in age > 18 years old and admission for high rate AF or atrial flutter requiring urgent/emergency cardioversion. A documented or suspected allergy or intolerance to midazolam or propofol represented the only exclusion criterion. No patient was excluded based on comorbidities or concomitant diseases in order to reflect “real-life” population data.

The study conforms to the Declaration of Helsinki and was performed in accordance with the Consolidated Standards of Reporting Trials (CONSORT) standards and national recommendations by competent authorities (Supplementary Table 1). The protocol was approved by the internal review board of our institution (University Hospital “Ospedali Riuniti Umberto I - Lancisi - Salesi”). Written informed consent was gained from all patients.

### Peri-procedural assessment

An accurate medical history was recorded from all patients in order to assess cardiovascular risk factors such as hypertension, diabetes, dyslipidaemia, family history and smoking habit. In addition, we recorded the patients’ comorbidities and known cardiovascular diseases. We also took note of the drug history and the number of previous cardioversions, electrical or pharmacological, that the patients had undergone. We performed routine blood tests, a 12 lead ECG and an echocardiogram for each patient.

### Cardioversion

**Fig. 1:**
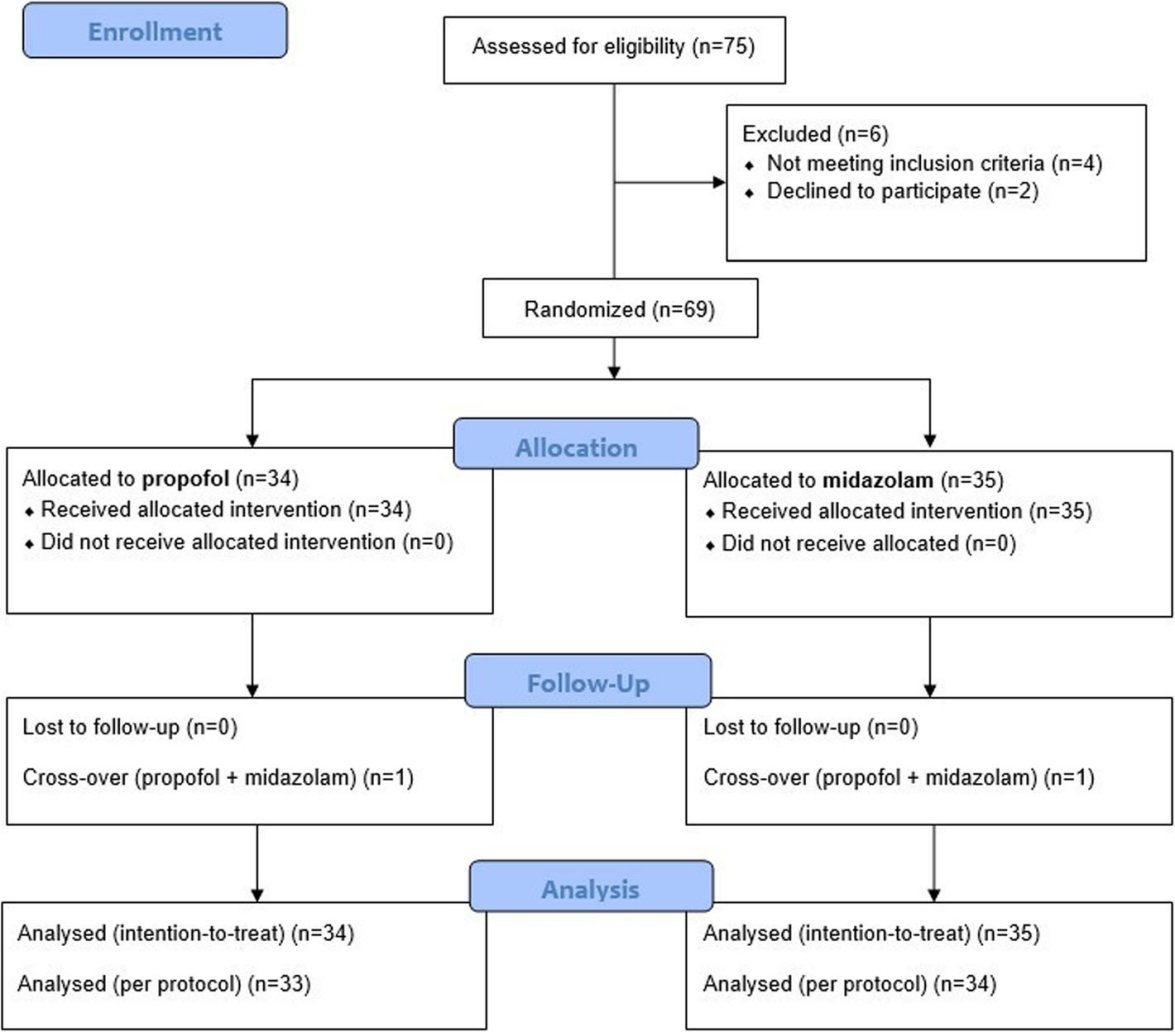
The CONSORT flow diagram detailing enrolment procedure

All enrolled patients were randomized in a 1:1 fashion into the propofol or midazolam group. The CONSORT flow diagram is showed in Fig. 1.

With regards to the propofol group, the procedure was carried out with the assistance of the anaesthesiologist who administered propofol 1 mg/kg followed by 0.5 mg/kg every 3 min until satisfactory sedation (Ramsay sedation scale > 4) [15]. The cardiologist delivered the shock through a manual external defibrillator (Zoll M-Series, Zoll Medical Corporation, Chelmsford, MA, USA).

In the midazolam group, both procedural sedation and DCC were carried out by the cardiologist who administered a starting dose of midazolam 3 mg, followed by 2 mg every 2 min until satisfactory response (Ramsay sedation scale > 4) [15]. The anaesthesiologist was readily available during the whole procedure in order to intervene if deemed necessary.

A maximum of three shocks were delivered, following a step up published protocol [16]: a first 150 J shock with manual paddles in antero-apical position. If not effective, a second shock of 200 J was delivered keeping the paddles in the same position. If both shocks failed to restore sinus rhythm, a third 200 J shock was delivered with adhesive patches in the anteroposterior position.

One-hundred percent oxygen supplementation and saline infusion were provided to each patient during the procedure.

At the end of the procedure, 1 mg of flumazenil was administered to the patients in the midazolam group in order to revert the effects of the benzodiazepine.

When awake, all patients were asked to rate the pain and distress on a scale going from no pain to the worst possible pain and distress according to a visual analogue scale (VAS).

A 12-lead ECG was performed at the beginning and at the end of the procedure in order to assess the restoration of sinus rhythm and all vital status parameters (blood pressure, heart rate, saturation) as well as delay, length of procedure, length of monitoring and hospitalization were registered.

### Endpoints

The primary safety endpoint was a composite of death and the following peri-procedural adverse events requiring medical intervention: bradycardia (defined as heart rate < 50 bpm for at least 30 s), severe hypotension (defined as systolic blood pressure < 80 mmHg), severe hypoxia (defined as oxygen saturation < 85%), stroke, transient ischaemic attack and need for orotracheal intubation.

Other safety endpoints included the variation of blood pressure, heart rate and oxygen saturation registered before induction, before delivery of the shock, after the shock and after recovery. Tolerability of the procedure was assessed using the VAS.

Efficacy was assessed based on the number of very early recurrences (within 2 h) and early recurrences (within 24 h). All patients discharged before 24 h from the Emergency Department were called by phone the day after the procedure in order to confirm stable sinus rhythm. Time-related issues (delay and length of the procedure, monitoring time and length of hospitalization) were also considered.

Finally, we analysed the cost-effectiveness of the procedure carried out by the cardiologist alone with midazolam, compared to procedural sedation with propofol and anaesthesiologist assistance. Direct total costs were defined as the total of personnel costs, material costs and hospitalization costs. For a more detailed definition of costs calculation please refer to our previous study [13]. Indirect costs were not taken into account.

### Statistical analysis

Quantitative variables were checked for normality by the Kolmogorov–Smirnov test, and described as mean and standard deviation (if normally distributed) or median and first to third quartile (if not normally distributed). ANOVA adjusted by age and sex was used to compare normally distributed quantitative variables. Kruskal– Wallis ANOVA adjusted by age and sex was used to compare non-normally distributed quantitative variables. Categorical variables were described by absolute number and assessed by using χ2 analysis. General linear model for repeated measures was used to assess time-dependent changes of blood pressure, heart rate and oxygen saturation. As one patient per group ended up having both interventional drugs administered (Fig. 1), an intention-to-treat analysis was adopted.

Being a feasibility study, a formal, a-priori, sample size calculation was not performed. However, considering an expected composite endpoint rate of 18% from our pilot study [14], a population of ≥34 subjects in each group would have a > 80% power to show that the primary safety endpoint (composite of death and the following peri-procedural adverse events) for the midazolam group was non-inferior to the control group, considering a preserved fraction (i.e. the fraction of the expected main effect that was estimated as clinically relevant) of 50% [17] (α = 0.05, one-tail test). Therefore, an increase of more than 50% of the composite endpoint rate in the midazolam group when compared to the propofol group would have been deemed as inferior.

SPSS 25.0 for Windows (SPSS Inc., Chicago, IL, USA) was used for statistical analysis. Values of *p* < 0.05 were taken as statistically significant.

## Results

### General characteristics

Sixty-nine patients were consecutively enrolled in our study, 61 of which (84.4%) had atrial fibrillation and eight (11.6%) were admitted with atrial flutter. General characteristics of the population, including risk factors, comorbidities, underlying heart disease, echocardiographic parameters and previous arrhythmic episodes are described in Table 1.

**Table 1:** General characteristics

Variable	Total population (*n* = 69)	Propofol group (*n* = 34)	Midazolam group (*n* = 35)	*P*-value
Male gender (n, %)	45 (65.2)	20 (58.8)	25 (71.4)	.272
Age (years)	66.5 ± 12.0	67.9 ± 12.6	65.1 ± 11.4	.322
BMI (kg/m2)	28.5 ± 5.2	28.0 ± 3.6	29.1 ± 6.1	.713
Hypertension (n, %)	49 (71.0)	26 (76.5)	23 (65.7)	.325
Diabetes (n, %)	7 (10.1)	4 (11.8)	3 (8.6)	.660
Dyslipidaemia (n, %)	28 (40.6)	13 (38.2)	15 (42.9)	.696
Smoking habit (n, %)	16 (23.2)	7 (20.6)	9 (25.7)	.614
COPD (n, %)	14 (20.3)	7 (20.6)	7 (20.0)	.321
CRI (n, %)	4 (5.8)	3 (8.8)	1 (2.9)	.289
Thyroid disorders (n, %)	13 (18.8)	6 (17.6)	7 (20.0)	.319
CHA2DS2-VASc score (median, 1st-3rd)	2 (1–3)	3 (2–3)	2 (1–3)	.649
Haemoglobin (g/dl)	13.6 ± 3.3	13.5 ± 3.1	13.7 ± 3.7	.806
eGFR (ml/min)	85.7 ± 33.6	83.4 ± 37.3	88.0 ± 29.7	.581
Sodium (mEq/l)	134.2 ± 29.1	135.3 ± 24.4	133.0 ± 33.4	.749
Potassium (mEq/l)	4.0 ± 1.0	4.0 ± 0.9	3.9 ± 1.0	.696
BNP (pg/ml)	257.2 ± 86.4	245.4 ± 71.5	269.2 ± 99.9	.276
Type of heart disease:				
Hypertensive	40 (58.0)	19 (55.9)	21 (60.0)	.729
Ischemic	11 (15.9)	7 (20.6)	4 (11.4)	.298
Valvular	5 (7.2)	3 (8.8)	2 (5.7)	.618
Idiopathic dilatative	2 (2.9)	1 (2.9)	1 (2.8)	.486
Lone AF	11 (15.9)	4 (11.8)	7 (20.0)	.350
Echographic characteristics:				
LAD (mm)	29.6 ± 18.1	30.4 ± 19.0	29.0 ± 17.3	.749
LVEDD (mm)	49.2 ± 8.7	49.3 ± 7.7	49.2 ± 9.8	.965
LVESD (mm)	30.1 ± 6.4	30.4 ± 6.4	31.6 ± 6.4	.487
LVEF (%)	44.4 ± 15.0	44.7 ± 14.9	44.2 ± 15.4	.930
AAD at enrolment:				
Flecainide (n, %)	15 (22.7)	7 (21.9)	8 (23.5)	.873
Propafenone (n, %)	4 (6.1)	2 (6.2)	2 (5.8)	.933
Amiodarone (n, %)	5 (7.6)	3 (9.4)	2 (5.9)	.592
β-blockers (n, %)	31 (47.0)	15 (46.9)	16 (47.1)	.988
CCBs (n, %)	18 (27.3)	10 (31.3)	8 (23.5)	.482
Anti-platelet (n, %)	15 (21.7)	7 (20.6)	8 (25.0)	.669
Anticoagulant (n, %)	52 (75.4)	26 (76.5)	26 (74.3)	.869
Previous AF episodes (median, 1st-3rd)	2 (1–3)	2 (0–3)	2 (1–3)	.353
Previous DCC (median, 1st-3rd)	0 (0–1)	0 (0–2)	0 (0–1)	.267
Previous PC (median, 1st-3rd)	0 (0–1)	0 (0–1)	0 (0–1)	.844

*AAD* Anti-arrhythmic drug, *AF* Atrial fibrillation, *BMI* Body mass index, *BNP* Brain natriuretic peptide, *CCB* Calcium channel blocker, *COPD* Chronic obstructive pulmonary disease, *CRI* Chronic renal impairment, *DCC* Direct-current cardioversion, *eGFR* Estimated glomerular filtration rate, *LAD* Left atrial diameter, *LVEDD* Left ventricular end-diastolic diameter, *LVESD* Left ventricular end-systolic diameter, *LVEF* Left ventricular ejection fraction, *PC* Pharmacological cardioversion

### Primary composite safety endpoint

Eighteen patients (26.1%) met the composite safety endpoint (Table 2). No deaths, neurological sequelae or need for orotracheal intubation were reported. All adverse events in the midazolam group were all managed successfully by the cardiologist with no need for anaesthesiology support or advanced airways management. Procedural sedation was optimally tolerated with propofol as well as with midazolam as all reported a VAS value of zero after DCC.

**Table 2:** primary safety endpoint

Adverse events	Propofol group (*n* = 34)	Midazolam group (*n* = 35)	*P*-value
Total (n, %)	10 (29.4)	8 (22.9)	.535
Bradycardia (n, %)	3 (8.8)	5 (14.3)	.479
Severe hypotension (n, %)	5 (14.7)	3 (8.6)	.426
Severe hypoxia (n, %)	2 (5.9)	0 (0)	.145
Neurologic event (n, %)	0 (0)	0 (0)	–
Orotracheal intubation (n, %)	0 (0)	0 (0)	–
Death (n, %)	0 (0)	0 (0)	–

### Secondary safety endpoints

**Fig. 2:**
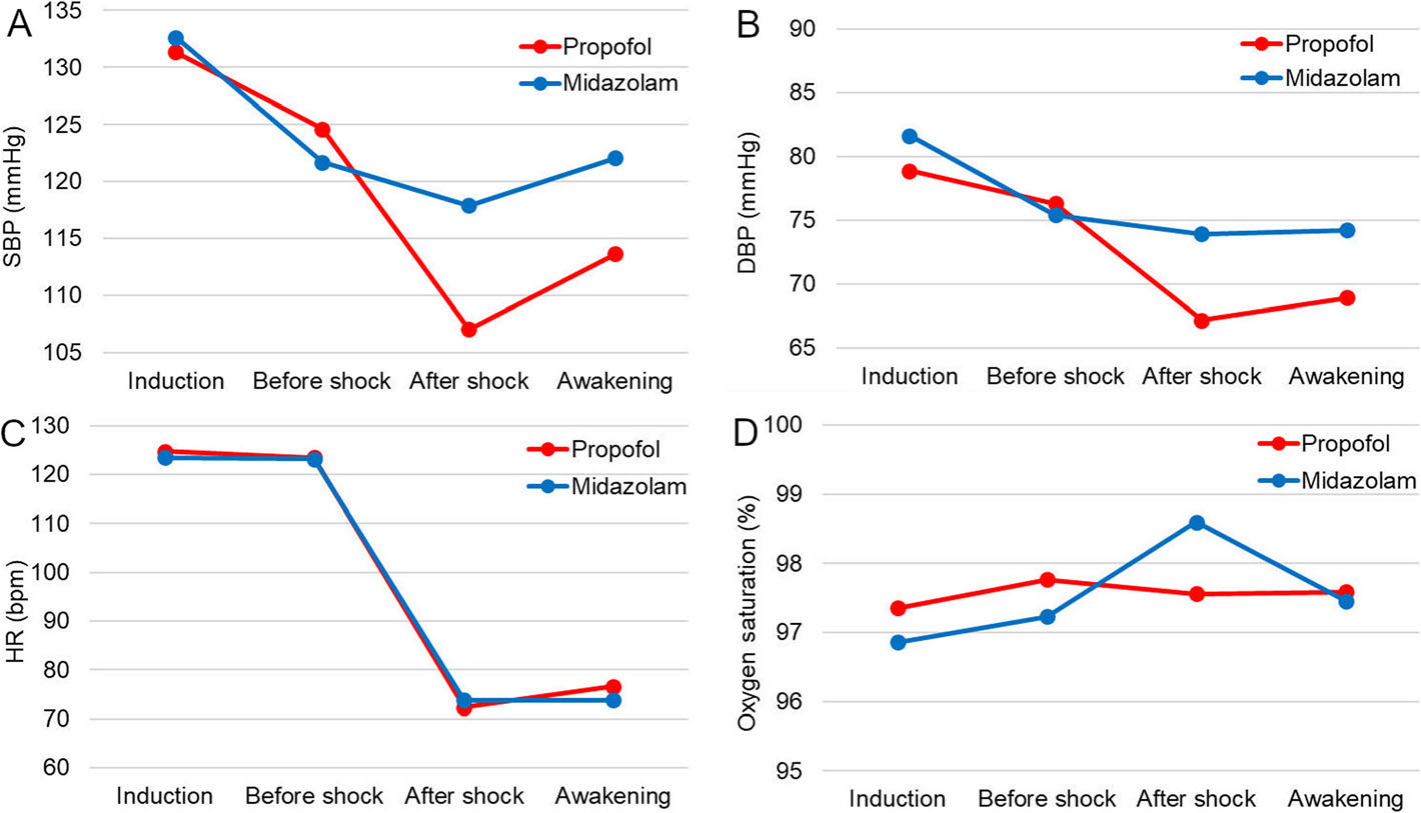
Time-related variations of systolic blood pressure, (**a**) diastolic blood pressure, (**b**) heart rate (**c**) and oxygen saturation (**d**) during cardioversion

With regards to the variation of vital status parameters patients in the propofol group experienced a greater, although not statistically significant, decrease in systolic (*p* = .336) and diastolic (*p* = .347) blood pressure than those treated with midazolam (Fig. 2a-b). The trends for both heart rate and oxygen saturation were similar in both group (*p* = .885 and *p* = .392 respectively, Fig. 2c-d). As the figure shows, heart rate plummeted after the shock, indicating sinus rhythm being restored at a lower rate. In the midazolam group, no patient experienced re-sedation after flumazenil administration.

### Efficacy endpoint

Ten recurrences were registered in the first 24 h. Of those, six were registered in the propofol group (four very early, two early) and four were registered in the midazolam group (two very early and two early), with no significant difference between groups (*p* = .692).

### Time-related endpoints

**Fig. 3:**
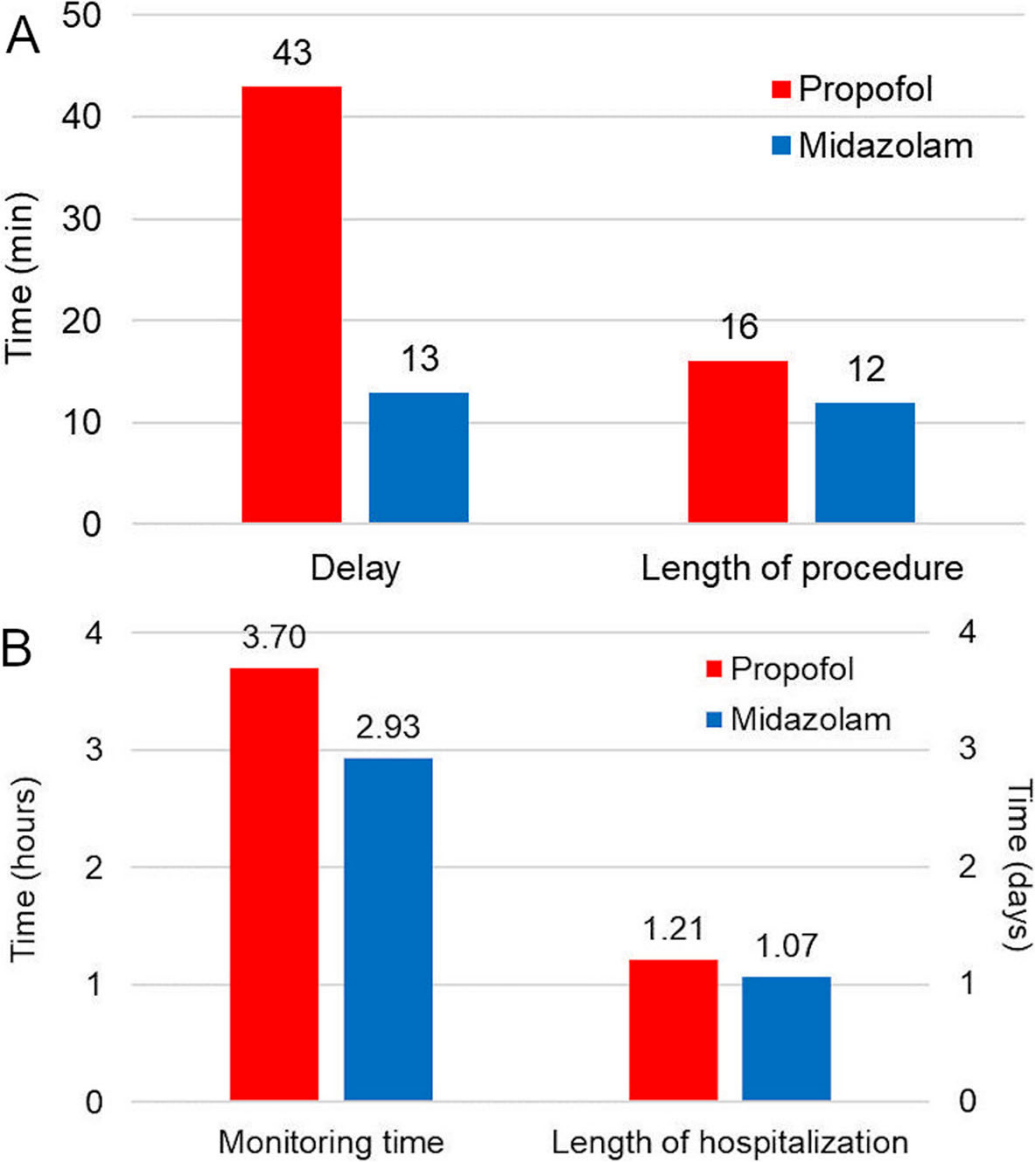
Differences in procedural times: (**a**) procedural delay and procedural length; (**b**) monitoring time and total in-hospital time

DCC randomized to propofol took on average 4 min more to perform (16 ± 7 min vs. 12 ± 5 min; *p* = .013) and were burdened by a 30 min higher delay which was needed to get more personnel ready (43 ± 11 min vs. 13 ± 4 min; *p* = .016; Fig. 3a). Monitoring time did not differ between the two groups (3.7 ± 2.0 h for propofol vs. 2.9 ± 1.4 h for midazolam; *p* = .107), nor did median hospitalization length (1.2 days for propofol vs. 1.1 days for midazolam; Fig. 3b).

### Costs analysis

The median medical cost was of 16.4 € for the midazolam group and 47.8 € for the propofol group (*p* < .001) and was mainly driven by an increased delay and lack of coordination between the cardiologist and the anaesthesiologist. In terms of material costs, the median cost in the midazolam group was higher than the propofol one as the former implied the use of flumazenil (83.7 € vs. 78.8 €, *p* < .001). Hospitalization costs in the midazolam group added up to a median of 28.1 € and 48.7 for the propofol group (*p* = .022). The total median cost of urgent/emergency DCC with midazolam was estimated to be 129.0 € (1st-3rd quartiles 114.6–151.6) and 195.6 € (1st-3rd quartiles 147.3–726.7) with propofol (*p* < .001).

## Discussion

Procedural sedation plays a key role in many procedures carried out for arrhythmia related issues such as device implant, electrophysiological studies, catheter ablations [18] and DCC [6]. As these procedures can be painful, an adequate level of sedation not only is required but can also reduce the pain-related catecholamine surge therefore preventing the recollection of an unpleasant experience [13]. Sedation has been described as a continuum including progressive stages: minimal sedation or anxiolysis, moderate sedation/analgesia, deep sedation/analgesia and general anaesthesia [5].

The results of the present study confirm that procedural sedation with midazolam could be a viable alternative to the propofol-based protocol, even in an emergency/urgency setting. Both drugs showed a similar safety profile as no patient experienced death, neurological events or needed orotracheal intubation. The number of adverse events in the midazolam group were fewer than those experienced in the propofol group and more importantly all of them were easily managed by the cardiologist alone with no need of intervention or assistance by the anaesthesiologist. On a note, the number of adverse events and the recurrence rates during emergency/urgent DCC were higher when compared to events in elective procedures [13]. This result may be secondary to the fact that in an emergency/urgent setting a higher variation of vital status parameters may be seen, leading to patients that are more labile and prone to adverse events.

Regarding recurrences, the difference was not statistically significant between the two groups. However, it is important to underline that the rate of recurrence could be different in patients who undergo urgent or emergency DCC compared to elective procedures. In fact, arrhythmia recurred in 14% of our patients, more than what usually expected in elective DCC [19]. Potential hypotheses for the higher rate of recurrence could include high variation of vital parameters, higher rate of comorbidities and absence of pre-treatment with antiarrhythmic drugs in most of our patients.

The procedure carried out with the benzodiazepine was well tolerated as demonstrated by the fact that all patients scored a zero on the VAS scale. We feel that such a result is not only secondary to the sedation obtained with midazolam, but also partly related to the anterograde amnesia caused by the drug. Moreover, as described above, while oxygen saturation and heart rate had a similar trend in both study groups, patients who received propofol experienced a more important drop in systolic blood pressure than in those in the midazolam group. This data confirm the effect of propofol as a peripheral vasodilator, therefore causing a greater impact on haemodynamic parameters [20].

In addition to this, the results clearly show how the length of the procedure and the associated delays can be shortened by using midazolam instead of propofol. We believe that the use of flumazenil as an antagonist to midazolam is the key factor contributing to a reduced procedural time. Flumazenil has been described to be a safe and effective reversal agent [21] and allows a shorter recovery period. However, given the difference in the half-life between midazolam and its antagonist, concerns have been expressed regarding potential re-sedation [22]. Our data support the safety of flumazenil, with no patients experiencing re-sedation during monitoring time or after discharge.

Our findings show that the use of midazolam can reduce costs by decreasing the time spent for the procedure itself and by having fewer operators needed to perform the procedure, as the cardiologist can safely manage both sedation and shock delivery. Standardizing a protocol which does not imply the need for anaesthesiologist support appears cost-effective.

### Limitations

Some limitations of the present trial deserve to be discussed. Firstly, double-blinding was deemed technically unfeasible due to the different number of physicians involved, the different intravenous formulations and the different titration protocols. Second, differences in experience and expertise of the cardiologists with midazolam could have provided an unintentional bias, albeit very difficult to measure. Third, the lower-than-expected difference in the composite endpoint between the two groups leaves open the chance of a type II error, even if of minor magnitude from a clinical point of view. However, it must be stressed out that the rate of severe complications was too low to allow for any firm conclusion regarding safety, being this a feasibility study with a relatively small sample size. Fourth, the present results could be difficult to extrapolate in other clinical settings, especially in those countries in which cardiologists are trained in advanced airways management or in which DCC are not primarily managed by the cardiologist [10].

Finally, we found that one patient who regularly took benzodiazepines for insomnia had built a tolerance which limited our possibility of using midazolam and had to be crossed over to propofol. Nonetheless, we also had one patient who probably metabolized propofol too quickly and as a consequence midazolam had to be used for sedation. However, we feel that this examples strongly confirm the need of finding easily manageable, safe and well tolerated options for procedural sedation.

## Conclusions

A cardiologist-only approach to procedural sedation with midazolam could represent a feasible and well tolerated option for DCC even in the emergency/urgency setting. Adverse events were managed by the cardiologist in both groups with no need for intervention by the anaesthesiology team. The length of procedure was shortened when midazolam was used and fewer delays were registered, thus potentially leading to reduced costs.

## Supplementary information

**Additional file 1: Supplementary Table 1.** CONSORT 2010 checklist of information to include when reporting a randomised trial.

## Data Availability

The dataset supporting the conclusions of this article will be available upon request to the corresponding author.

## Abbreviations

AF: Atrial fibrillation
DCC: Direct current cardioversion
ECG: Electrocardiogram
VAS: Visual analogical scale
